# Outcome of patients with acute pancreatitis requiring intensive care admission: A retrospective study from a tertiary care center of Pakistan

**DOI:** 10.12669/pjms.345.15575

**Published:** 2018

**Authors:** Faraz Shafiq, Muhammad Faisal Khan, Muhammad Ali Asghar, Faisal Shamim, Muhammad Sohaib

**Affiliations:** 1 Faraz Shafiq, Department of Anaesthesiology, The Aga Khan University, Karachi, Pakistan; 2 Muhammad Faisal Khan, Department of Anaesthesiology, The Aga Khan University, Karachi, Pakistan; 3 Muhammad Asghar Ali, Department of Anaesthesiology, The Aga Khan University, Karachi, Pakistan; 4 Faisal Shamim, Department of Anaesthesiology, The Aga Khan University, Karachi, Pakistan; 5 Muhammad Sohaib, Department of Anaesthesiology, The Aga Khan University, Karachi, Pakistan

**Keywords:** Pancreatitis, Critical care, Retrospective, Tertiary, Outcome

## Abstract

**Background and Objective:**

Acute pancreatitis (AP) is an inflammatory disease. Patients presenting with severe disease may require intensive care unit (ICU) admission. Factors predicting mortality and morbidity need to be identified for improving outcome. The objective of this study was to see the outcome of these patient presented to single center over a period of ten years. The secondary objective was to identify the factors responsible for adverse outcome.

**Methods:**

The medical records of adult patients from year 2006 to 2016 requiring ICU admission for AP were reviewed retrospectively. The data was collected on the predesigned Performa for patient’s demographic, etiology, severity of disease and reason of ICU referral. Besides this physiological and biochemical parameters at time of arrival in ICU were also recorded. Management aspects related to disease course including the ICU related complications were also recorded. The outcome was predicted on the basis of mortality and length of stay (LOS) in ICU and hospital.

**Results:**

Total 85 patients were identified of having AP requiring ICU admission. 56% of these cases were referred from emergency. Mean Ranson score (RS) was 2.6 and 2.7, at and after 48 hours of admission. Necrosis was present in 48% of cases. Mean APACHE-II score was 23. Sepsis was the commonest complication in ICU. The median LOS in ICU and hospital was six and 12 days respectively. The overall hospital mortality was 52%, out of which 82% died in ICU. RS at admission and APACHE were correlated well with outcome. Similarly associations of factors like need of vasopressors, ARDS, pneumonia, sepsis and AKI requiring intervention were also related to mortality. Likewise development of necrosis or intra-abdominal hypertension showed increased mortality. Biochemical parameters serum blood urea nitrogen (BUN), P_H_ and serum glutamic-oxaloacetic transaminase were also directly linked to adverse outcome.

**Conclusion:**

AP patients requiring ICU admission represent severe form of disease. There is a need to develop protocol based care, which should be started immediately after hospital admission. This should have special focus on fluid resuscitation and nutritional therapy. Role of simple bed site parameters like BUN needs to be evaluated.

## INTRODUCTION

Acute pancreatitis (AP) is an inflammatory condition of pancreas, the worldwide incidence of which is 13 to 45/100,000.^1^ Ingestion of alcohol, gallstone and hypertriglyceridemia being the common etiological factors.^2^ The presentation of disease may vary from mild self-limiting course to a severe form requiring intensive care (ICU) admission for monitoring and organ support.^3^ ICU course may be complicated by factors like sepsis, multi-organ dysfunction syndrome (MODS), hospital acquired infections or acute kidney injury (AKI).^4^ The outcome really depends upon integrated health care services. As most of current literature is from advanced health care setup, where etiological factors and management protocols are much different. Hence, it’s important to identify disease pattern, course and outcome in our population. This would be helpful in standardizing care of these patients according to international guidelines.

The objective of study was to evaluate the outcome as judged by in hospital mortality for patients admitted to ICU with diagnosis of AP. The secondary objective was to evaluate the factors associated with adverse outcome.

## METHODS

The study was started after getting approval from hospital ethical committee. The Health information and management system was involved to retrieve medical record numbers of all adult patients (age 18-60 years) admitted in hospital with diagnosis of AP. It included the review of medical record of AP from year 2006 to 2016. Patients required ICU admission during the course of their disease were included, while patients having mild pancreatitis and managed else where were excluded from study. Total 186 patients were identified, out of which 104 were excluded and finally 85 patients were included in study protocol. Three primary investigators working as critical care physician collected the data on predesigned Performa. Each investigator was allocated with 63 number of patient’s files. They were supposed to review the relevant data on a predesigned Performa, which had information regarding patient’s demographic including the age, sex, BMI and associated comorbid conditions. The severity of AP was judged on the basis of first computed tomography (CT) findings and Ranson scoring (RS) system,^5^ at time and after 48 hours of hospital admission. The CT scans signs of edema/inflammation or necrotizing pancreatitis (NP) was also recorded. The nutritional status of patients at time of referral was also recorded. The disease course in ICU was evaluated on basis of referrals type, that is either from emergency department (ED), operation theatres or wards. The reasons for such referrals were also recorded which includes sepsis, acute respiratory distress syndrome (ARDS), acute kidney injury (AKI), multi-organ dysfunction syndrome (MODS), post cardio-pulmonary resuscitation (CPR). To predict the severity of physiological disturbance at time of arrival in ICU various biochemical parameters were recorded. It included the information regarding the status of Hemoglobin (Hb), hematocrit (HCT), platelet counts (PLT), white cell count (WBC), international normalized ratio (INR), serum Albumin, liver function test (LFT), serum electrolytes, blood urea nitrogen (BUN), serum creatinine (CR), serum lactate, serum amylase and lipase level, serum calcium level and arterial blood gas. Acute physiology and chronic health evaluation II (APACHE II) score was used to predict severity of disease after 24 hours of ICU admission.^6^ Need of mechanical ventilation, and vasopressor or renal replacement therapy (RRT) during ICU course was recorded. Possible complications that could occur during ICU course were also recorded. It included the documentation regarding occurrence of sepsis, ARDS, catheter related blood stream infection (CLABSI), pneumonia, cardiac arrest, renal failure, intra-abdominal hypertension or psudocyst formation. The management of AP in ICU was evaluated on the basis of conservative steps or any surgical/radiological intervention was required.

The outcome of these patients was declared on basis of ICU mortality and ICU LOS, and hospital mortality and hospital LOS. ICU mortality was defined as number of deaths happened during ICU stay. It was said to be to earlier if occurred within 48 hours, and delayed if happened after 48 hours of admission. ICU LOS was calculated by evaluating the number of days patient spend in unit. Hospital mortality was defined as death occurred after discharge from the ICU with in a period of 30 days. Hospital LOS was calculated by number of days patient spend in the hospital after discharged from ICU.

Statistical analysis was performed using Statistical packages of social sciences (SPSS ver-19, Inc., Chicago, IL, USA). The normality of numeric distribution was determined by Kolmogorov-Smirnov or Shapiro-Wilk test. Categorical point estimation was reported in term of frequency and percentage while numeric point estimation was reported in term of mean (standard deviation) or median (25^th^-75^th^ percentile). Unpaired t test or Mann whiney U test was used to compare mean difference of outcome between survived and non-survived groups. Chi-square test or fisher exact test was applied to compare proportion difference of outcome between survived and non-survived groups. A p value ≤ 0.05 was considered (two sided) statistical significant.

## RESULTS

**Fig.1:**
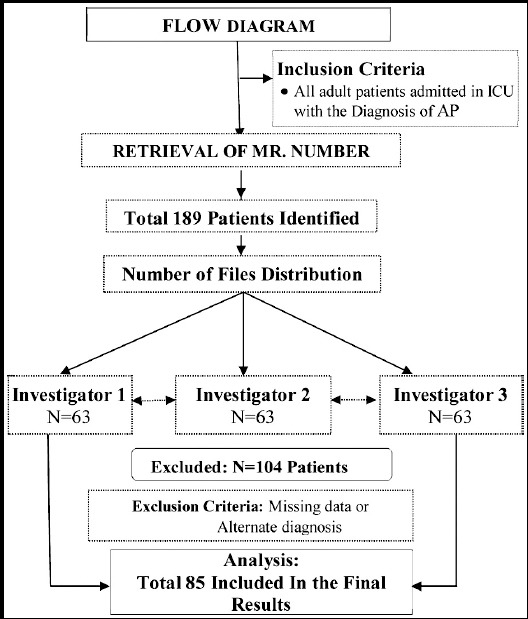
Flow Diagram.

Out of 189 patients 85 were fulfilling the inclusion criteria and required ICU admission. Mean age of patients was 49 years, out of which 55% were males and 45% females. Most of these patients were obese having mean BMI of 30.3 Kg/m_2_. Association of comorbid conditions showed predominance towards hypertension (44.7%) and diabetes mellitus (30.6%). Presence of gall stones (54%) was the main etiological factor which lead to AP. Other causes were alcohol consumption (13%) and idiopathic (15%). 56% of patients were admitted directly from ED, while rest of them were referred from special care unit (22%), general ward (6%) and operating room (15.3%). The sepsis and respiratory failure were the commonest reason of referrals, which were present in 45% and 57% of patients respectively. Other causes were AKI, MODS and Post CPR (Fig.1). The severity of AP as depicted by RS showed mean score of 2.6 at admission, and 2.7 after 48 hours. CT scan showed presence of edema /inflammation in 50% of cases while necrosis was there in 48%. After admitting to ICU, 82% of these patients required mechanical ventilation. Similarly 80% required inotropic support. 15% of these patients required renal replacement therapy. The mean APACHE II score of patients was 23. The disease course was complicated with intra-abdominal hypertension (22%), pancreatic abscess (9%) and pseudo cyst formation (12%). 86% of these patients were treated medically. The surgical intervention was required in 12%, while only 2.4% of patients required radiological intervention. Significant number of patients (49%) were nil per orum (NPO) at time of admission to ICU unit, while enteral nutrition was started in 44% and total parenteral route (TPN) was used in 8% of patients (Table-I).

**Table-I:** Diagnosis, Severity, ICU course and complications related to AP.

Variables	Point Estimate	Range / Percentage
***Severity of Acute Pancreatitis***		
***Ranson Score***		
At Admission	2.61±1.12	1-6
After 48 hours	2.7±1.35	0-6
APACHE-II	23.1±7.70	1-42
Vasopressor Required	68	80 %
CRRT Required on Admission	13	15.30%
***Complication of Acute Pancreatitis***		
Intra-abdominal HTN	19	22.40%
Pancreatic abscess	8	9.40%
Retroperitoneal abscess	1	1.20%
Pseudo cyst	10	11.80%
Encephalopathy	11	12.90%
Other	2	2.40%
None	34	40%
***CT Finding***		
Edema/ Inflammation	42	49.40%
Necrosis	41	48.20%
Other	6	6%
***Feeding***		
NPO	42	49.40%
Enteral	38	44.70%
PPN	1	1.20%
TPN	7	8.20%

Point Estimate are presented as mean ± SD, Range and n(%)

The biochemical parameters at ICU admission showed mean HCT of 33, WBC counts of 17 x1000, Cr of 1.7 with BUN of 34, serum lactate of 3.5 and HCO3 of 17. The mean P_H_ on arterial blood sampling was 7.32 (mean base deficit of -5.51). Mean serum lipase and amylase level were significantly elevated that is 729 and 465 respectively. There was a significant derangement in serum glutamic-pyruvic transaminase (SGPT) and serum glutamic-oxaloacetic transaminase (SGOT).

**Fig.2:**
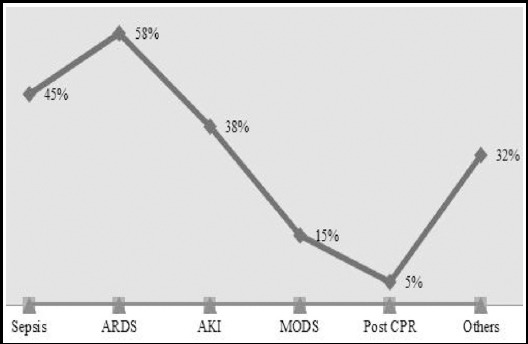
Reasons of admission to intensive are unit (ICU).

The median LOS at the ICU was six days, which was complicated by sepsis in 32% of patient, AKI (30%), CLABSI in 3.5%, Pneumonia (20%), ARDS (20%) and cardiac arrest in 15% of patients. For significant number of patients (22%), code do not attempt resuscitation (DNR) status was decided. The median H-LOS was 12 days (Table-I). The overall in hospital mortality was 52% (n=44) out of which 82% (n=36) died during their stay in ICU, while 18% (n=8) had death in ward. Early ICU mortality that is death, which occurs within a period of 48 hours was 22% (n=8) and 78% of these (n=28) died after 48 hours of admission (Fig.2).

**Fig.3:**
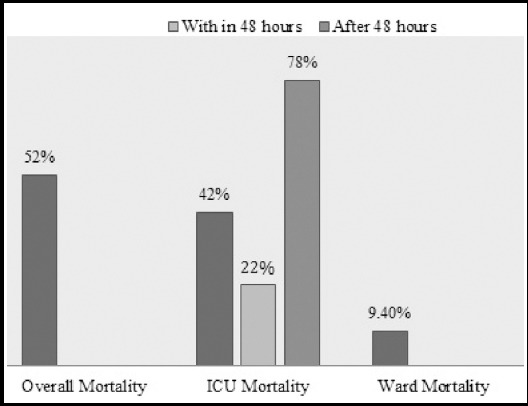
Overall mortality in patient with AP.

Comparison of various parameters between survivors and non-survivors was also done. The factors associated with adverse outcome (P value < 0.005) were the RS at the time of admission to hospital and APACHE II score after arrival in ICU. Use of vasopressor, development of sepsis, MODS and ARDS were also the predictors of mortality. If patient developed cardiac arrest or those who declared DNR were associated with higher mortality. Presence of necrosis in the gland or if pancreatitis course worsen because of intra-abdominal hypertension lead to increased mortality. Similarly the BUN, SGOT and Serum PH level at the time of admission in the ICU were associated with adverse outcome. (Table-III).

**Table-II:** Management, ICU related complication, mortality and stay status of the acute pancreatitis patients.

Variables	Point Estimate	Range / Percentage
***ICU Related Complications***		
Sepsis	27	31.8%
Renal failure	25	29.4%
CLABSI	3	3.5%
Pneumonia	17	20%
Cardiac arrest	13	15.3%
ARDS	17	20%
DNR	19	22.4%
***Length of Stay in Days***		
ICU - Median [Q3-Q1]	6[11-3]	1-73
Hospital - Median [Q3-Q1]	12 (18-7)	1-78

Point estimate are presented as median [IQR] and n (%)

**Table-III:** Comparison of various variables between Non survivors and survivors.

Variables	Non-Survived (n=44)	Survived (n=41)	P-Value
***Reason Of ICU admission***			
ARDS/Respiratory failure	32 (72.7%)	17 (41.5)	0.004
MODS	11(25%)	2 (5%)	0.01
***Ranson Score***			
At admission	3.03	2.06	0.0005
APACHE II	27.15	18.62	0.0005
***ICU management***			
Norepinephrine	28 (63.6%)	10 (24.4%)	0.0005
RRT	11(25%)	2 (2%)	0.014
***ICU related complication***			
Cardiac arrest	13(29.5%)	0(0%)	0.0005
ARDS	14(31.8%)	3(7.3%)	0.005
DNR	16(36.4%)	3(7.3%)	0.001
***CT findings***			
Necrosis	27(66%)	14(34%)	0.012
Feeding			
NPO status	29(66%)	13(31%)	0.002
Enteral	14(32%)	24(59%)	0.013
***Complications related to AP***			
Intra-abdominal hypertension	15(34%)	4 (10)	0.007
***Laboratory parameters***			
Serum BUN	39	24	0.003
SGOT	112	62	0.000
PH	7.30(7.36-7.21)	7.37(7.45-7.29)	0.009

Results are presented as n (%) and median [IQR]

## DISCUSSION

The management of AP should follow a stepwise approach. Evaluating severity of disease is important not for predicting mortality, but also helpful in utilization of resources. Even with advancement in health care facilities, disease still carries a huge burden in terms of cost, complications and outcome. The lack of local data in the presence of inadequate health care facilities and management protocols make our patients vulnerable to have adverse outcome. Pal KM et al reported mortality of around 5-40% for NP,^7^ while Alvi AR and colleagues reported it to be somewhere between 17-39%.^8^ However, data for both of these studies was collected in patients having NP and were managed in surgical ward.

Studies have shown the impact of etiological variables^9^ on the outcome of AP but this was not observed in this study. The work done by Juneja D et al.^10^ showed that pancreatitis associated with alcohol consumption represents the severe form. According to their results, chances of conversion to NP were quiet high. However, this was not depicted in our results. Data emerging from our region shows gallstone bring the commonest cause of AP.^11^ Gallstones were the cause in 46% of our patients. This might be related to cultural difference, limited availability and amount of alcohol consumption. The Cucher D *et al*.^12^ reported mortality of gallstone pancreatitis around 20% in advanced health care setup. But in our part of the world gallstone pancreatitis seems to be a severe form of disease and associated with higher mortality.

We did not identify the impact of age, weight and sex on outcome of these patients. Significant numbers of patients in our study were referred directly from the ED. Almost half of them (45-55%) had ongoing sepsis and ARDS, which were correlating well with the pathophysiological aspects of disease.^13^ Moreover its also reflecting that disease process is severe to start with, as evident by organ system involvement. Adequate fluid resuscitation is mainstay of therapy at this stage. It reverts not only systemic inflammatory response but also halt the progression of AP to NP. At arrival to ICU, almost 38% of our patient had AKI. This was associated with mean BUN of 34, Cr of 1.7, Base deficit of -5.5 and Lactate of 3.5. This shows clear gaps in pre ICU care of these patients, where they might not adequately resuscitated. A recent multicenter retrospective trial conducted in patients having AP by Yamashita T et al also showed mortality benefit in patients had resuscitation with large volume of fluid.^14^ The association of AKI is also reported to be a risk factor for increasing morbidity, mortality and economic cost^15^ of treatment. Though AKI itself was not reported to be an independent risk factor of adverse outcome in this study but there was a significant difference in mortality of patients who need RRT versus who did not require (25% vs. 5%, P= 0.014).

Mean APACHE II score of patients was 23. The predicted mortality at this score is around 70%. Earlier, the study conducted in same ICU observed actual mortality of around 60%^16^ at this score range. Here, the score is showing same convincing results in terms of predicting outcome. There was a statistical significant difference in mean APACHE II score between the survivors and non-survivor (P= 0.0005). The predictive value of Ranson scoring system is questionable in our study. The mean score in our study population was 2.6 and 2.7 at and after 48 hours of admission. According to this criterion the predicted mortality should be around 15% for score between 0-2, and 15% for score between 3-4. The reliability of Ranson score has already been questioned in various studies.^17^ Cho JH *et al*.^18^ reported its sensitivity of around 85% and specificity of 44.3% respectively. However, score at admission was significantly associated with mortality in our study (P= 0.0005).

Around 49% of our patients showed edema/inflammation on and 48% showed necrosis of varying degree on initial CT scan report. Like previous studies, the presence of necrosis showed significant association with mortality. Though most of our physicians prefer to start antibiotic empirically^19^ but still the septic complications were on the top of list followed by renal failure and pneumonia. Both ARDS and MODS were significantly associated with the adverse outcome. The mortality in our study was also directly linked to fasting status. About 49% of patients were NPO at time of arrival in ICU, as preference is always given to naso-jejunal (NJ) over naso-gastric (NG) feed. Putting NJ tube needs radiological expertise, a common reason of delay in starting nutrition in these patients. The level of BUN, Cr, and metabolic acidosis at time of presenting ICU was significantly associated with mortality. Recently some studies have focused the utilization of individual tests like BUN and Cr. The study results by Bu WU et al confirmed the significance of monitoring BUN in terms of mortality prediction and as reliable surrogate marker to aid fluid resuscitation.^20^ Besides this both of these parameters have proven to have accurate in comparison to complex scoring system.^21^ It’s very important for us to look at these biochemical parameters considering the cost and availability.

### Limitations

There are few limitations associated with our study. Considering the retrospective nature of study various parameters were collected at certain point in time and definitely there is a chance that we might have missed some relevant clinical information. Similarly we don’t know what was the disease severity in terms of systemic inflammatory response at time of presenting to the hospital. Moreover we don’t know what regime was used for fluid resuscitation.

## CONCLUSION

The results of this retrospective data showed high morbidity and mortality of AP requiring ICU admission. We recommend the implementation of care bundles right from the start of hospital admission. Amongst which fluid resuscitation and nutritional protocol should have prime importance. More over there is an urgent need to evaluate the efficacy of simple bed site parameters like BUN and Cr in terms of predicting outcome and guiding level of care.

### Author`s Contribution

**FS:** Proposal write up, ERC approval, looking after the data collection, write-up.

**MFK:** Proforma designing and data collection.

**AA and FS:** Data collection.

**MS:** Data entry, statistical analysis and submission to the journal.

## References

[1] Yadav D, Lowenfels AB (2013). The epidemiology of pancreatitis and pancreatic cancer. Gastroenterology.

[2] Hasibeder WR, Torgersen C, Rieger M, D€unser M (2009). Critical care of the patient with acute pancreatitis. Anaesth Intensive Care.

[3] Tenner S, Baillie J, DeWitt J, Vege SS (2013). American College of Gastroenterology guideline:management of acute pancreatitis. Am J Gastroenterol.

[4] Pavlidis P, Crichton S, Lemmich SJ, Morrison D, Atkinson S, Wyncoll D (2013). Improved outcome of severe acute pancreatitis in the intensive care unit. Crit Care Res Pract.

[5] Ranson JH, Rifkind KM, Roses DF, Fink SD, Eng K, Localio SA (1974). Objective early identification of severe acute pancreatitis. Am J Gastroenterol.

[6] Knaus WA, Draper EA, Wagner DP, Zimmerman JE (1985). APACHE II:a severity of disease classification system. Crit Care Med.

[7] Pal KM, Kasi PM, Tayyeb M, Mosharraf SM, Fatmi Z (2012). Correlates of morbidity and mortality in severe necrotizing pancreatitis. ISRN Surg.

[8] Alvi AR, Sheikh GM, Kazim SF (2011). Delayed surgical therapy reduces mortality in patients with acute necrotizing pancreatitis. J Pak Med Assoc.

[9] Barauskas G, Ignatavicius P, Vitkauskienee A, Pundzius J, Dambrauskas Z (2015). Impact of etiology on course and outcomes of severe acute pancreatitis. Medicina.

[10] Juneja D, Gopal PB, Murthy RV, Malhotra P (2010). Outcome of Patients With Alcoholic Pancreatitis Admitted to Intensive Care Unit: A single center experience. Ann Gastroenterol.

[11] Iqbal M, Malik M, Perveen S (2015). Morbidity and Mortality in Acute Pancreatitis. J Surg Pak (Int).

[12] Cucher D, Kulvatunyou N, Green DJ, Jie T, Ong ES (2014). Gallstone pancreatitis:A review. Surg Clin North Am.

[13] Du XG, Chen XM, Gan H, Li ZR, Wen YJ, Wang XC (2011). Continuous blood purification ameliorates RhoA-mediated endothelial permeability in severe acute pancreatitis patients with lung injury. Int J Artif Organs.

[14] Yamashita T, Horibe M, Sanui M, Sasaki M, Sawano H, Goto T (2018). Large volume resuscitation for severe acute pancreatitis is associated with reduced mortality. A multicenter retrospective study. J Clin Gastroenterol.

[15] Petejova N, Martinek A (2013). Acute kidney injury following acutepancreatitis: A review. Biomed Pap Med Fac Univ Palacky Olomouc Czech Repub.

[16] Naved S, Siddiqui S, Khan F (2011). APACHE-II Score Correlation with Mortality and Length of Stay in An Intensive Care Unit. J Coll Physicians Surg Pak.

[17] Papachristou GI, Muddana V, Yadav D, O'Connell M, Sanders MK, Slivka A (2010). Comparison of BISAP, Ranson's, APACHE-II, and CTSI scores in predicting organ failure, complications, and mortality in acute pancreatitis. Am J Gastroenterol.

[18] Cho JH, Kim TN, Chung HH, Kim KH (2015). Comparison of scoring systems in predicting the severity of acute pancreatitis. World J Gastroenterol.

[19] Khan A, Khan S (2010). Antibiotics in acute necrotizing pancreatitis-perspective of a developing country. J Pak Med Assoc.

[20] Wu BU, Bakker OJ, Papachristou GI, Besselink MG, Repas K, van Santvoort HC (2011). Blood urea nitrogen in the early assessment of acute pancreatitis:an international validation study. Arch Intern Med.

[21] Mounzer R, Langmead CJ, Wu BU, Evans AC, Bishehsari F, Muddana V (2012). Comparison of existing clinical scoring systems to predict persistent organ failure in patients with acute pancreatitis. Gastroenterology.

